# SU-8 free-standing microfluidic probes

**DOI:** 10.1063/1.4975026

**Published:** 2017-02-14

**Authors:** A. A. Kim, K. Kustanovich, D. Baratian, A. Ainla, M. Shaali, G. D. M. Jeffries, A. Jesorka

**Affiliations:** 1 Department of Chemistry and Chemical Engineering, Chalmers University of Technology, Göteborg 412 96, Sweden; 2 Department of Physiology and Pharmacology, Karolinska Institutet, Stockholm 17177, Sweden; 3 Department of Microtechnology and Nanoscience, Chalmers University of Technology, Göteborg 412 96, Sweden

## Abstract

We present a process for fabrication of free-standing SU-8 probes, with a dry, mechanical release of the final micro-devices. The process utilizes the thermal release tape, a commonly used cleanroom material, for facile heat-release from the sacrificial layer. For characterization of the SU-8 microfluidic probes, two liquid interfaces were designed: a disposable interface with integrated wells and an interface with external liquid reservoirs. The versatility of the fabrication and the release procedures was illustrated by further developing the process to functionalize the SU-8 probes for impedance sensing, by integrating metal thin-film electrodes. An additional interface scheme which contains electronic components for impedance measurements was developed. We investigated the possibilities of introducing perforations in the SU-8 device by photolithography, for solution sampling predominantly by diffusion. The SU-8 processes described here allow for a convenient batch production of versatile free-standing microfluidic devices with well-defined tip-geometry.

## I. INTRODUCTION

There is increasing evidence that data collected on single cells, in contrast to ensemble averages, often provides a more complete picture of cellular processes relevant to healthy and pathological states.^1,2^ Progress is, however, limited by the current lack of techniques and methods for tracking, isolating, manipulating, and analyzing single cells. There is a pressing need for the development of novel technologies that specifically target individual cells. Sophisticated high throughput techniques for suspended cells are already quite developed, using microfluidic devices for transport and manipulation.^3,4^ Comparable practical solutions for adherent cultures and tissues are very scarce. Free-standing microfluidic probes are of great interest, as they provide flexibility in terms of positioning and operation, and are thus compatible with traditional cell culturing techniques.^5,6^ They allow for a considerable variety of applications in the context of life science experiments.

In hydrodynamic flow confinement (HFC) devices, liquids are injected and aspired simultaneously by applying positive and negative pressures to a number of channels with exits located at the tip of a probe, resulting in a recirculation, or flow confinement, zone in between the device and the surface. The confinement zone is self-sustaining, and can be repositioned together with the device. Analytes of interest can be delivered and collected in a contact-free, selective manner. We have previously reported on a multifunctional pipette (MFπ), which uses HFC in combination with flow switching to provide local chemical stimulation to a selected target, enabling a wide range of experimental setups, including single-cells, tissue slices, and muscle fibers.^7–10^

However, some challenges still remained to be addressed, for instance, the observed constraints of the switching speed, imposed by the elastic walls of polydimethylsiloxane (PDMS), the fabrication limits of the pipette tip geometry, the manufacturing scalability of the PDMS replica molding process, and the difficulty of integrating additional functional components, such as electrodes. These challenges are a consequence of the material properties and processing requirements of PDMS. These problems prompted us to investigate the possibility of employing the negative photoresist SU-8, which has been widely used in microsystems and coating applications,^11–15^ for a scalable process with options for integration of additional features.

SU-8 is a high contrast photo-patternable epoxy that exhibits excellent mechanical strength, thermal and chemical stability along with good coating and planarization properties. SU-8 can be directly patterned into a structure of interest with high aspect ratio, allowing for robust and scalable fabrication processes. Multilayer structures can be achieved in SU-8 by successive processing of several photoresist layers applied on top of each other.^16,17^ The presence of unreacted epoxy groups on the surface of the polymer allows for a direct bonding of wafers hosting pre-fabricated SU-8 structures.^18,19^ In this way, 3D devices containing hollow channels can be constructed, and released from the wafer by a number of techniques. The high rigidity of SU-8 makes it a suitable material for needle-like probes as it can be inserted into tissues, while still having a sufficient elasticity to tolerate accidental contact with solid surfaces. In contrast to PDMS, SU-8 does not require any post-processing, such as cutting or punching of the material, in order to shape the devices or introduce fluid ports. Probe tips in the micrometer range as well as millimeter-sized port openings can be defined using photolithography, resulting in planar, very thin devices. This is of advantage in microscopy studies, since it minimizes shadow-casting by the device, which we typically observe with the millimeter-thick PDMS structures.

In this paper, we present an alternative microfabrication process for HFC devices, employing the SU-8 multilayer approach. The characteristic structure of these devices is a pointed tip with 2–3 adjacent channel exits, which allow for positioning of the tip close to a surface bound object. The focus of our fabrication route is, besides exploiting the up-scaling capabilities of a photolithographic process, on greatly improving the tip geometry, in comparison to the results of the previously reported PDMS replica molding/post-processing route.

**FIG. 1.**
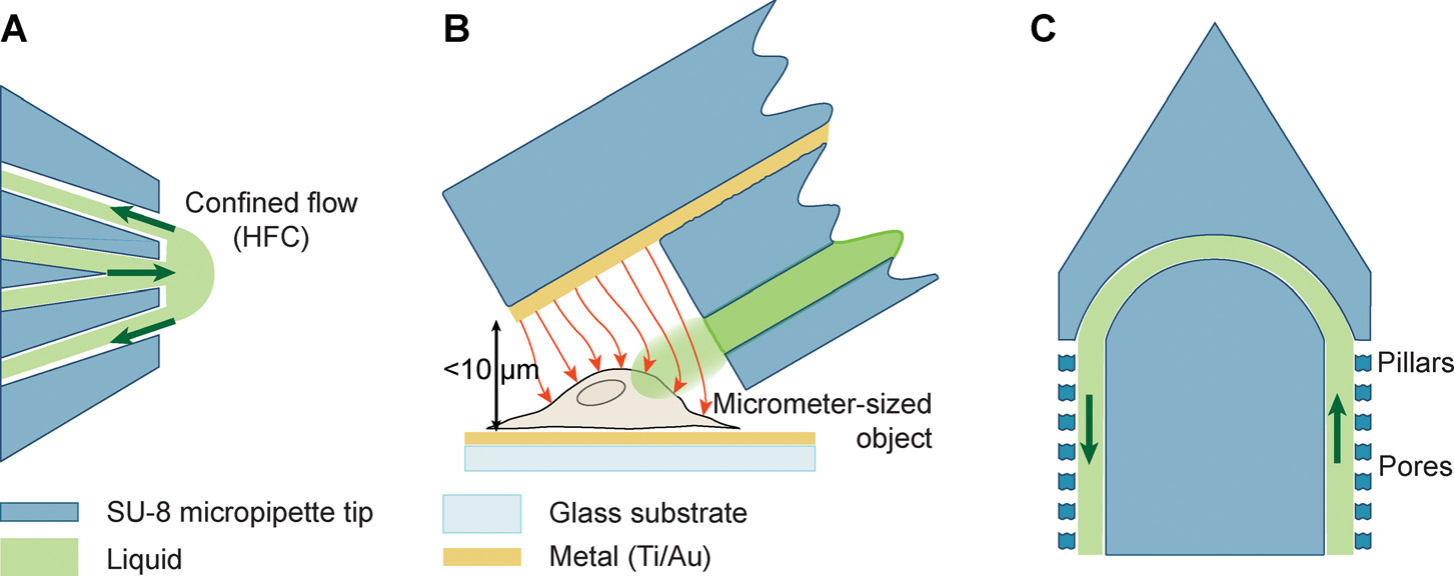
Schematic representations of the probe designs. The green arrows indicate the direction of the liquid flow, and the red arrows indicate the electric field lines. (a) Top view of a microfluidic probe for confined solution delivery. (b) Side view of a probe with integrated thin-film electrodes, illustrating impedance measurements on a micrometer-sized object, e.g., a microbead or a biological cell. (c) Top view of a probe with perforated walls for microdialysis.

We use a single-sided thermal release tape as sacrificial layer, which enables a solvent-free, facile mechanical final detachment of the structures. Thermal release tape is a polyester-based tape, typically available in cleanroom environments, as the double-sided version is often used as a convenient temporary adhesion layer in various processes. The use of thermal release tape was inspired by the separation problems experienced with other sacrificial layers such as overhead transparency film,^20^ Kapton^®^ polyimide film in combination with an adhesive,^21,22^ polyester,^23^ PDMS,^24^ or Mylar^®^ (Ref. 25) sheets. Other reports on the use of metallic layers such as aluminum^26^ and copper^27^ in combination with wet etching also have critical disadvantages, similar to polystyrene in combination with toluene^28^ and oxide layers.^29^ These processes use solvents and plasmas that not only contaminate the produced microchannels, but also tend to swell, or permanently modify the SU-8 surface, affecting its material integrity, wetting properties, and biocompatibility. The thermal release tape allows for dry release of microfluidic devices with a high yield and reduced process complexity, providing larger batches and consequently, reduced fabrication time per device. In addition, the thin feature of the devices improves usability in optical microscopy studies, as it facilitates the positioning close to a substrate surface. Since the possible application range for HFC devices in the life sciences is large, three practical implementations were developed: (i) a microfluidic probe, (ii) a probe with integrated metal thin-film electrodes, and (iii) a probe with perforated walls for microdialysis, schematically illustrated in Figure 1.

The devices were fabricated on the wafer scale with up to 26 devices per 4″ wafer, with the exception of (iii). Characterization of the probes was performed with respect to the requirements defined for applications in hydrodynamically confined solution delivery (i), impedance spectroscopy (ii), and microdialysis (iii). The focus for the microdialysis probe was on the possibilities of introducing additional structures into the device body by photolithography, here in the form of perforations for solution sampling by diffusion, and eventually to hold the microdialysis membrane (diffusion barrier).

## II. MATERIALS AND METHODS

### A. Microfabrication of SU-8 microfluidic probes

**FIG. 2.**
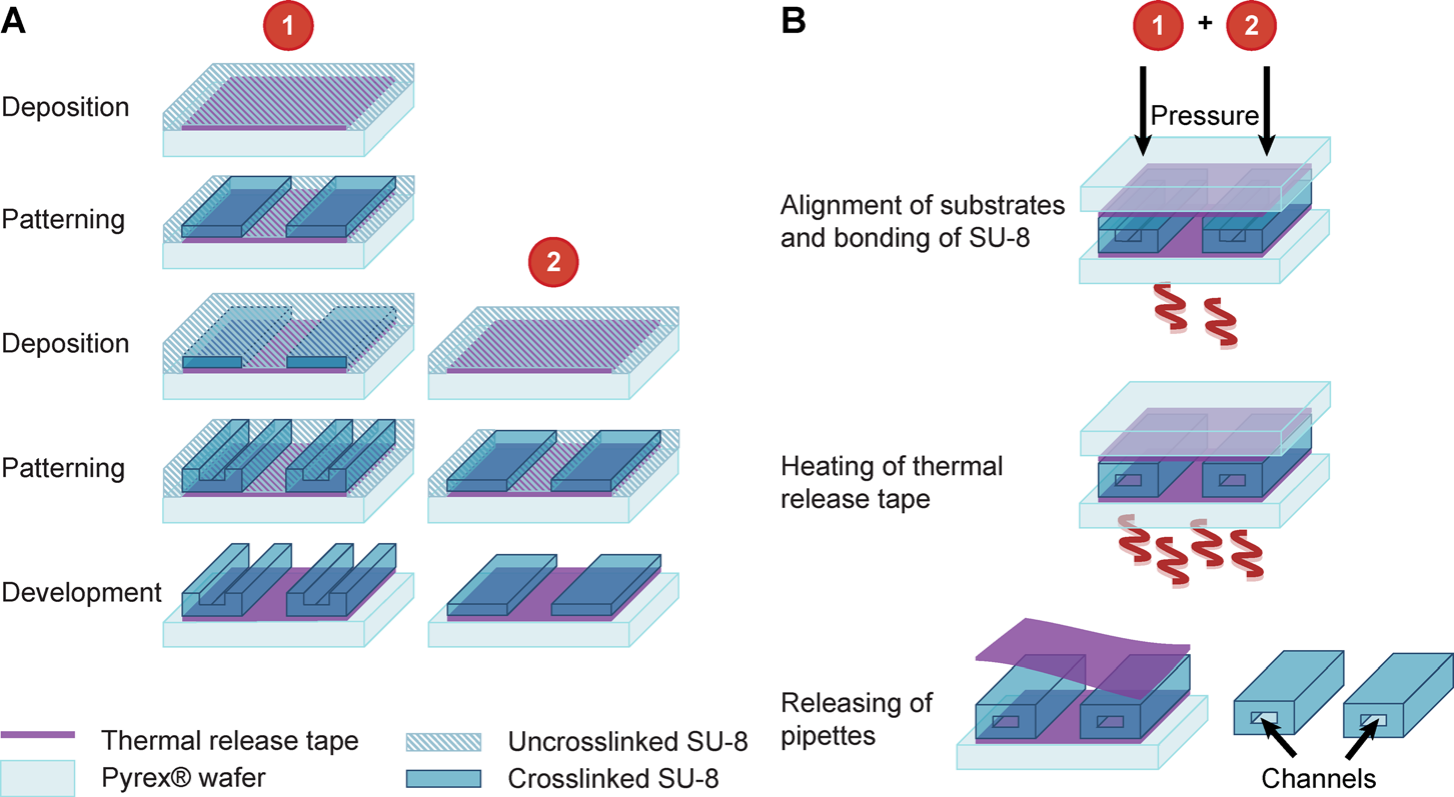
Schematic illustration of the fabrication procedure of the SU-8 microfluidic probes, using thermal release tape as sacrificial layer. (a) Simultaneous photolithographic processing of two wafers (identified by the numbered circles), and (b) bonding of the two wafers and the dry release procedure of the microdevices.

Fabrication of the SU-8 devices was performed at the Nanofabrication Laboratory at MC2, Chalmers University of Technology. The process, which is illustrated in Figure 2, has its foundation in an earlier report.^21,26^ Two wafers are processed in parallel (see Figure 2(a)), with microfluidic channels defined on one wafer, and the lid on the other. The processing parameters used to fabricate the free-standing microfluidic probes can be found in the supplementary material S1.

The procedure begins with shaping the single-sided thermal release tape (Revalpha 3195VS or 3195HS, Nitto Denko Corp, Japan, courtesy of Teltec GmbH) so that the photolithographically defined alignment marks will be outside of the tape, directly on the wafer. This simplifies the eventual optical alignment of the two wafers prior to bonding. The tape is then carefully applied on Pyrex^®^ wafers (Ø 4″, University Wafer, USA). In order to improve the adhesion between the thermal release tape and SU-8, a minimal exposure to oxygen plasma can be performed (1–3 s, 10 sccm, 50 W, 250 mTorr in PlasmaTherm Reactive Ion Etcher).

**FIG. 3.**
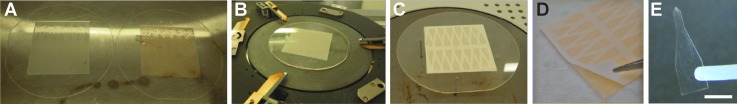
Photographs of the dry, mechanical release of the SU-8 devices. (a) Top and bottom layers of the devices on individual 4″ Pyrex^®^ wafers, prior to bonding. (b) Aligned and assembled wafers ready for bonding. (c) Bottom wafer with bonded devices between tapes, after thermal release of the top wafer. (d) Removal of the top layer of the thermal release tape. (e) Final SU-8 device after removal from the tape before the hard-bake. Scale bar 5 mm.

SU-8 (MicroChem, USA) was deposited on top of the substrate, and visible air bubbles were removed with a disposable pipette (BLE Spinner). A soft-bake was performed after spin-coating of SU-8, first at 65 °C, and then at 90 °C. Prior to exposure, the SU-8 was allowed to rest for a few minutes in order to avoid building up of stress. The exposure dose was corrected for the substrate and the thickness used (SÜSS MicroTec MA6). The SU-8 was post-exposure baked, first at 65 °C, and then at 90 °C under-baked (by reducing the temperature by 5 °C) to prevent complete crosslinking of the polymer for subsequent layers and bonding.^21^ After the substrate was allowed to cool down slowly to room temperature, a subsequent SU-8 layer was spun on top, with the spinning speed corrected for the material underneath. The layer was soft-baked at 65 °C, and then at 90 °C. Acetone was used to remove the edge beads, and to reveal the alignment marks of the first SU-8 layer underneath. The first layer of SU-8 was used to define the overall shape of the tips, along with providing mechanical support and the microfluidic circuitry defined in the second layer. The post-exposure bake was performed at 65 °C, and then 90 °C. The additional wafer, with a single SU-8 layer was processed in parallel, which later served as the top layer in the bonding process. SU-8 was developed in mr-Dev 600 (Micro Resist Technology GmbH, Germany) and checked using optical microscopy. Care has to be taken during the development, as agitation of the solvent can lead to devices detaching from the thermal release tape. After the development, the wafers were aligned using a SÜSS MicroTec MA6 system. The wafers were then transported to the substrate bonder (SB6, Süss MicroTec) and bonded at a temperature of 100 °C and an effective pressure (force/device surface area) of approximately 18 bars for 30 min (see Figure 2(b)). The wafers were gradually heated to the release temperature of the thermal release tape, and the sandwich removed from its mechanical support. Following that, the flexible tapes were removed to release the bonded SU-8 microfluidic probes. Particular care has to be taken in this step to avoid damaging of the microstructures. The photographs of the dry, mechanical release of the SU-8 microfluidic probes are shown in Figure 3.

**FIG. 4.**
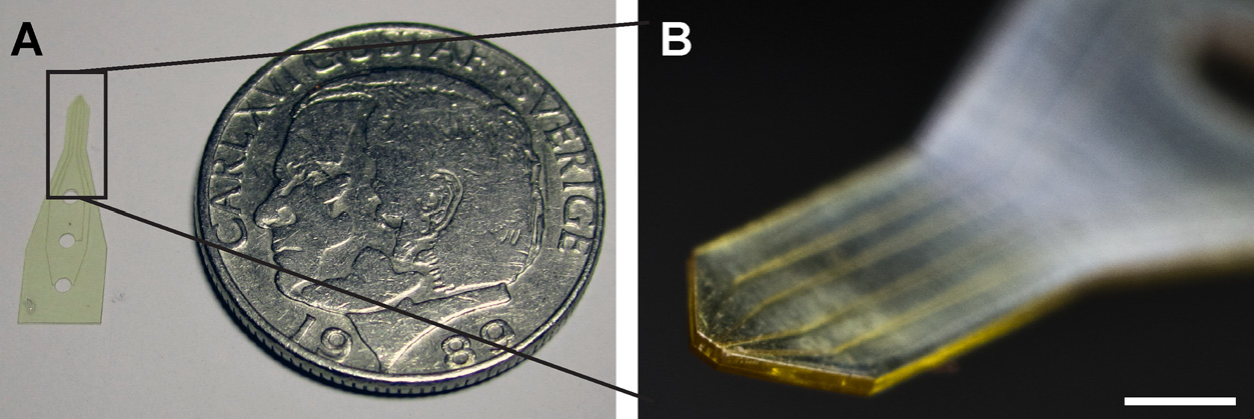
Photographs of SU-8 microfluidic probes. (a) A single SU-8 microfluidic probe next to a coin (Swedish crown, Ø 25 mm) and (b) a close-up of the SU-8 microfluidic probe tip. Scale bar 1 mm.

Following the release, the SU-8 microfluidic probes were hard-baked at 200 °C for 30 min with temperature ramping and cooling. The final result of the microfabricated pipette tips is shown in Figure 4.

### B. Fabrication of SU-8 probes with integrated metal thin-film electrodes

**FIG. 5.**
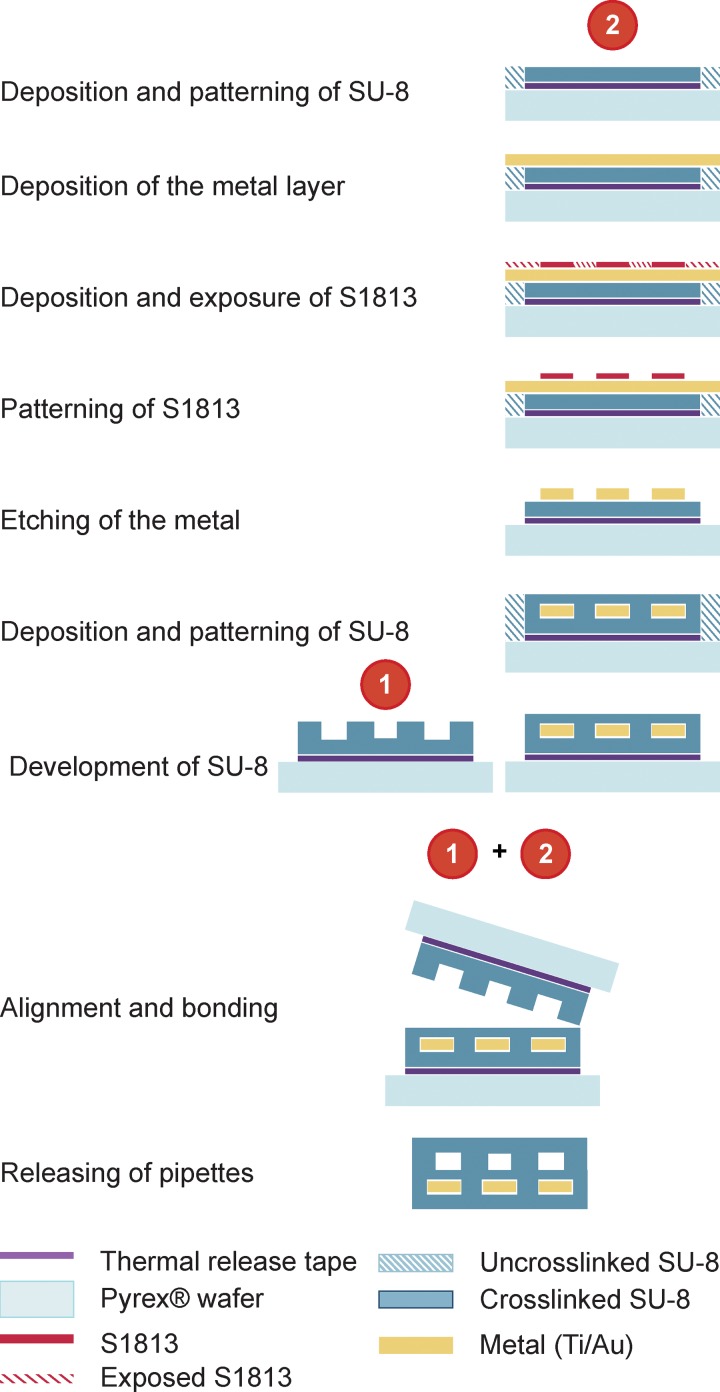
Schematic illustration of the extended fabrication process for integration of metal thin-film electrodes in SU-8 microfluidic probes.

The microfabrication process which integrates metal thin-film electrodes into the SU-8 structures is schematically illustrated in Figure 5. Since even a slight unevenness of the resist on the wafer during spin-coating can influence the final structure of the pipettes, it is significant to make a radial symmetry arrangement of the pipettes around the center of wafer. The design is such that the tips of the SU-8 devices are positioned towards the center of the wafer, i.e., where the resist is most even and precise control on multi-layer structuring could be achieved. Two wafers, one for the fluidics and one for the electrodes, were processed in parallel. The thin metal film was deposited on the patterned, yet undeveloped SU-8 layer for support prior to deposition.

After the metal film (Ti/Au) was deposited (Sputter FHR MS150), a positive photoresist (S1813) was spun on top and patterned to define the shape of the metal film, which was then chemically etched. Prior to bonding, another SU-8 layer was deposited and patterned, which acted both as the passivation layer for the electrodes and as the lid for the microfluidic structures. Post-baking was slightly shorter for the top exposed layers in order to guarantee a successful bonding with proper fluidic sealing. The duration of the bonding step was longer (∼45 min) due to the additional processing steps. Detailed processing parameters can be found in the supplementary material S1.

### C. Fabrication of SU-8 probes with perforated side walls

The fabrication process of the SU-8 probes with perforated side walls was adapted from the above-described process for the microfluidic tips. The first layer of SU-8 was applied and photo-patterned, which later served as a lid to provide the mechanical stability and seal the microfluidic channels of the probe. The second layer of SU-8 was spun and patterned with fluidic circuitry and pore structures. To minimize the effect of thermal release tape, the SU-8 film was left to relax at room temperature on a very flat-leveled surface for about an hour right after spin-coating.^13^ Long baking times at temperatures above the T_g_ (55 °C for uncured SU-8^30^) would also result in a superb flatness. The surface roughness of thermal release tape ultimately limited the pore size (see Section III).

### D. Interfacing of the SU-8 microfluidic probes

**FIG. 6.**
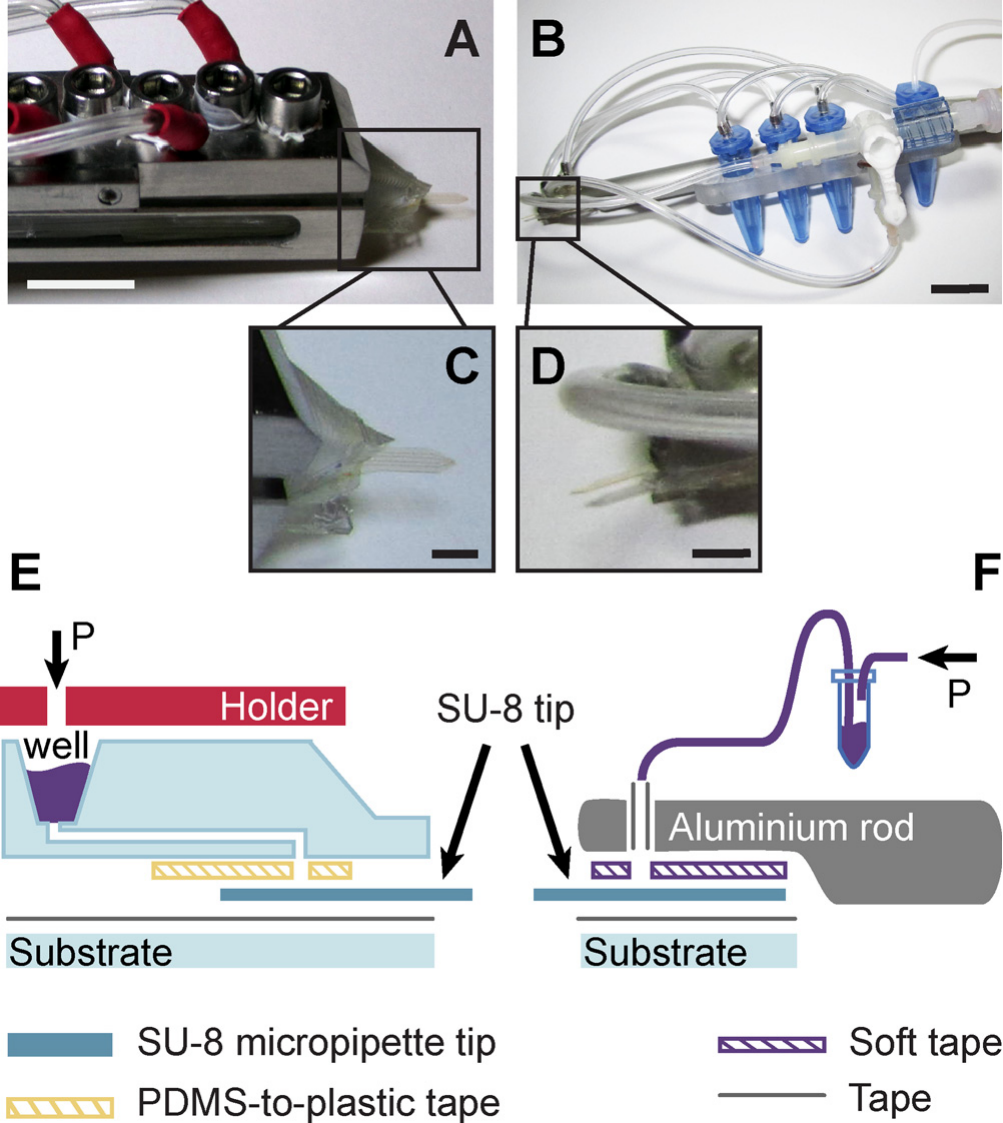
Interfacing schemes of the SU-8 microfluidic probes. Photographs of (a) PDMS body with integrated wells, and (b) aluminum rod with external Eppendorf tubes. (c) and (d) are the respective insets of the photographs, illustrating the SU-8 tips. (e) and (f) are the schematic side-views of the interfaces, respectively. The arrows indicate the connection to a pneumatic pressure source. The scale bars represent 10 mm in (a) and (b), and 3 mm in (c) and (d), respectively.

Fluid delivery experiments were performed to investigate the performance of the fluid delivery devices in an experimental environment that is typical for the multifunctional pipette. For that purpose, two different interfacing strategies were developed, which were used to deliver fluids to the device channels through circular ports: (A) double sided tape to a PDMS body containing internal reservoirs. This setup resembles closely the original multifunctional pipette design, and allowed an application of the dedicated holding manifold. (B) A clamp interface that connects the device ports through a rubber seal to a rod-mounted manifold that receives supplies through tubes and external reservoirs (Figure 6). Variant A, which produces a permanently connected hybrid device, is disposable, but has, due to the manual fabrication of shaped and perforated double-sided tape, greater time requirement for assembly, as compared to variant B), the quick-assembly interface based on a modified crocodile clamp. Both interfaces provide a stable performance, and differ mainly in their dead volume/sample requirement.

The characteristic differences between the two interfacing strategies, which are important in the context of microfluidic solution delivery, are outlined in Table I below.

**TABLE I.** Summary of the features of the two interfaces developed for the SU-8 microfluidic probes.

Interface	(A) PDMS-body/manifold	(B) Clamp/holding rod
Liquid storage	Integrated wells	External reservoirs (Eppendorf vials)
Capacity	30 *μ*l	>500 *μ*l
Dead volume	Low (nl)	High (*μ*l)
Assembly	Slow (h)	Fast (min)
Re-usability	Disposable	Maintenance

### E. Interfacing electronics and fluidics

**FIG. 7.**
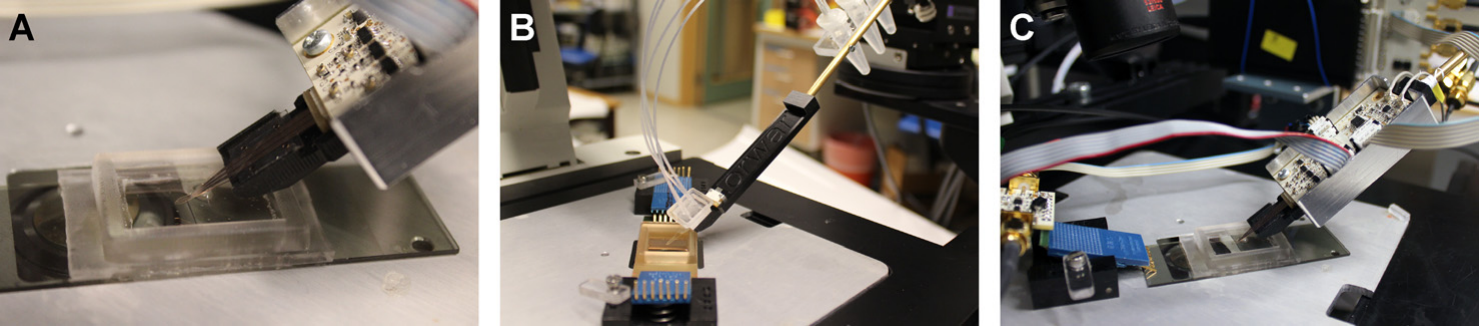
Interface schemes for the functionalized SU-8 microfluidic probe. (a) A PCB is connected to the probe tip (to interface the active and reference electrodes); (b) another PCB is connected to the substrate by a spring contact (to interface the counter electrode). The signals are transferred to the impedance analyzer (black box) that is connected to the computer. The fluidic connection between the pipette channels and the liquid reservoirs is arranged with a PDMS fluid-coupling piece in which the tubes are inserted. (c) Side-view of the interface.

A separate interfacing scheme, combining electric and fluid connections was developed for the SU-8 probes with integrated metal thin-film electrodes. The electric components for impedance measurements were provided by Prof. Martin Min (Tallin University of Technology, Estonia), along with the software. The fluidic interconnections were located close to the front of the SU-8 tips and the electric components further back. A complete setup of the interface developed for the integrated SU-8 probe is shown in Figure 7.

The interface for the impedance sensing consists of a seat structure at the front end, a pad for attaching a circuit board, and a metallic rod. The holder was fabricated out of a polyacetal black sheet (DELRIN^®^), which is an engineering thermoplastic used in the production of precision parts that require high stiffness, low friction, and excellent dimensional stability. A PDMS slab was used for supplying liquid via a set of external Eppendorf tubes, on one end relying on double-sided thermal release tape with precise openings as a gasket and a seal on the other. Electrical contacts were implemented as a plug-in extension for an electrical connecting socket (1.0 FPC Conn ZIF SMT from Elfa, Sweden).

### F. Characterization of the SU-8 microfluidic probes

Probe performance was characterized in terms of the time requirement for solution exchange, a main characteristic of the HFC devices with flow-switching capacity. SU-8 microfluidic probes with a thinner bottom layer (∼15 *μ*m) were simulated using the COMSOL Multiphysics software (see supplementary material S2). The devices were then fabricated, loaded with an aqueous fluorescent solution (1 mM fluorescein), interfaced, and positioned by means of a Narishige MH-3 micromanipulator on a microscope stage with the tip approximately 20 *μ*m above the sample surface. The fluorescent dye was illuminated at 488 nm using a total internal reflection fluorescence (TIRF) setup (in-house built setup using a Leica DM-IRB inverted microscope with 63 × 1.45 oil immersion TIRF objective), approximately 100 nm above the sample surface for characterization of surface solution exchange.

### G. Finite element model for probes with perforated side walls

The COMSOL Multiphysics software package was used in order to find the optimal flow conditions, and the required number of micropores for a recovery rate of analyte of ∼50%. A fully parametric model was developed to simulate the flow inside the device and diffusion across a range of micropores. The summary of the values used to design the system is supplied in the supplementary material S3. Channels of 20 × 20 *μ*m were used for guiding the perfusate. Each micropore was defined as 10 × 10 *μ*m while the diffusion constant of a molecule that passes through it was set to 2 × 10^−10^ m^2^/s. The diffusion coefficient of a molecule moving through the micropore to the external medium (water) was set to 10^−9^ m^2^/s. The corresponding flow rate inside the channels was set to 0.18 nl/min, which was chosen to accommodate the experimental limitations (cf. Section III D).

## III. RESULTS AND DISCUSSION

### A. Fabrication

We successfully established a fabrication process for layered microchannel devices in SU-8, using single-sided thermal release tape as the sacrificial layer. This reduces the fabrication time, eliminates the need for wet etching to chemically remove the sacrificial layer,^21,26^ and provides a clean, entirely mechanical release of the devices (cf. Figure 3). It generates well-reproducible tip geometries, and has also allowed us to significantly reduce the thickness of the tip from ∼1 mm (tapered) to 100 *μ*m, eliminating any shadow-casting effect under the microscope completely (Figure 4). Several SU-8 microfluidic probes were fabricated, tested in common optical microscopy application settings, and compared to earlier-presented PDMS pipette tips.^10^ However, the process does involve some practical limitations; the devices need to be larger than 200 *μ*m in size in order to reliably be released from the thermal release tape. Particular care needs to be taken during removal to avoid breaking of the devices. The sacrificial substrate has a relatively high surface roughness, which was characterized in terms of feature resolution. The minimum achievable feature size was found to be >5 *μ*m. Using an optical surface profiler (Wyko NT1100, Veeco), we estimated the average surface roughness and the root mean square to be 120 and 150 nm, respectively, for the released devices.

The fabrication process was typically performed on a 4″ Si wafer, and provided batches of 26 functional plain devices, or 12 metallized devices, respectively. Compared to the rather problematic Kapton and other films, which are glued to the substrates and require liquid immersion for lift-off, the heat release tape was effective for dry-releasing the sacrificial layer from the wafer upon heating above 150 °C. The pipettes are at this point still attached to the top side of the heat release tape, from where they are subsequently mechanically peeled off. The flexibility of the sacrificial layer is important for gentle release, and high yield of final devices. For the same purpose, the previously reported glue-on films need to be removed from the wafers by solvent action, as removal of the thin devices from a planar rigid surface poses a high risk of mechanical damage. Optical inspection of the tape-released devices revealed no residues or defects on the surfaces, and very well-defined channel exits (cf. Figure 4(b)). The softness of the thermal release tape means that much higher pressures are required in comparison to the previously reported process.^21,26^ The main drawback of this sacrificial layer is that the pressure calibration required for the substrate bonding process for different designs does not scale linearly with the surface area due to the softness of the thermal release tape, and requires optimization.

The time demand for the two fabrication processes outlined above is approximately 6–7 h, and 9–10 h, respectively, which compares very favorably with the PDMS device production, even though a single process (including time for mounting) requires ∼40% more time. The following sections (Sections III B–III D), details on the characterization, and example applications of the different devices (probes) are provided. Table II contains a summary of all parameters and dimensions that are important in the application contexts.

**TABLE II.** Comparison of characteristic dimensions and parameters of SU-8 and PDMS probes.

	SU-8 Tip	Metallized SU-8 tip	Microdialysis tip	PDMS MFπ^10^
Channel height × width (*μ*m)	30 × 30	40 × 30	20 × 20	30 × 30
Bottom thickness (*μ*m)	30 (15)^a^	40	30	15
Top structure (*μ*m)	30	180	30	>1000
Channel separation (*μ*m)	30	30	30	30
Width of the tip	400^b^	400^b^	-^c^	>400 (large variation)
Minimum feature size (*μ*m)	30	30	10 (pores)	30 (10)^d^
Switching time (ms)	<60	…	…	<100
Ports	Integrated	Integrated	Integrated	Post-processing
Tip shape	Integrated	Integrated	Integrated	Post-processing
Fluidic interface	Ports	Ports^e^	Ports	Device-integrated wells
Total fabrication time (h)	6–7	9–10	-^f^	5
Typical batch size (pcs)	26 (4″)	12 (4″)	-^f^	12 (6″), 5 (4″)^g^

^a^ Bottom layers of 15 *μ*m were implemented for comparison.

^b^ Could be as small as 200 *μ*m, limited by the mechanical release from the sacrificial layer.

^c^ No defined tip (no channel outlets).

^d^ Different device generations before and after performance optimization.

^e^ On-chip reservoirs are not possible due to the presence of electronic circuitry.

^f^ Not fabricated on the wafer scale.

^g^ Both 4″ and 6″ master wafers were used for replica molding.

### B. Characterization of the SU-8 microfluidic probes

**FIG. 8.**
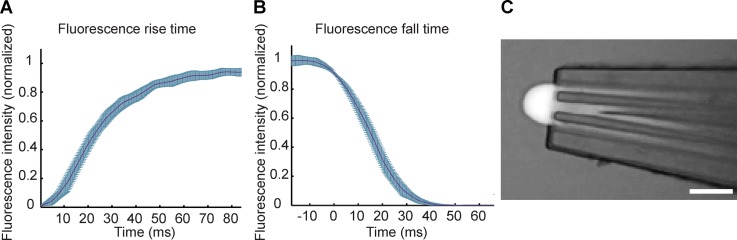
Characterization of the SU-8 microfluidic probes. Fluorescence (a) rise and (b) fall times, with A-type uncertainty, ±1 standard deviation (c) photograph of an SU-8 micropipette tip, illustrating the hydrodynamic flow confinement. Scale bar 100 *μ*m.

The interfaced SU-8 devices were capable of withstanding pressures between −0.95 and 0.95 bar (above typical operating pressures of ∼200 mbar), and their performance was characterized in terms of the time requirement for solution exchange, a main characteristic of the HFC devices with flow-switching capacity. A typical response plot is shown in Figures 8(a) and 8(b) with a rise time (10%–90% of maximum) of ∼50 ms and a fall time (90%–10% of maximum) of ∼30 ms for an applied pressure of 300 mbar and a negative recirculation pressure of 350 mbar. The hydrodynamic confinement of the fluorescein solution is shown in Figure 8(c) (see supplementary material S4 for more details). The results indicate faster switching speeds in comparison to the previously reported MFπ with switching speeds in the range of 100 ms,^10^ and the discrepancy is attributed to the elasticity of PDMS.

### C. Metallized SU-8 probes for impedance spectroscopy

Platforms for impedance spectroscopy have shown a great potential for analysis of liquids^31,32^ and micrometer-sized objects,^33–35^ leading to recent interest in the development of free-standing impedance probes.^36^ The SU-8 process allows for an easy integration with other common fabrication processes, specifically thin film deposition techniques. The above presented fabrication route was extended further in order to incorporate the deposition and precise patterning of gold electrodes on selected surface areas of the SU-8 microfluidic probes. The low frequency impedance response can potentially be useful for determining the position of the device relative to objects on the surface, which would not require an optical microscope.

**FIG. 9.**
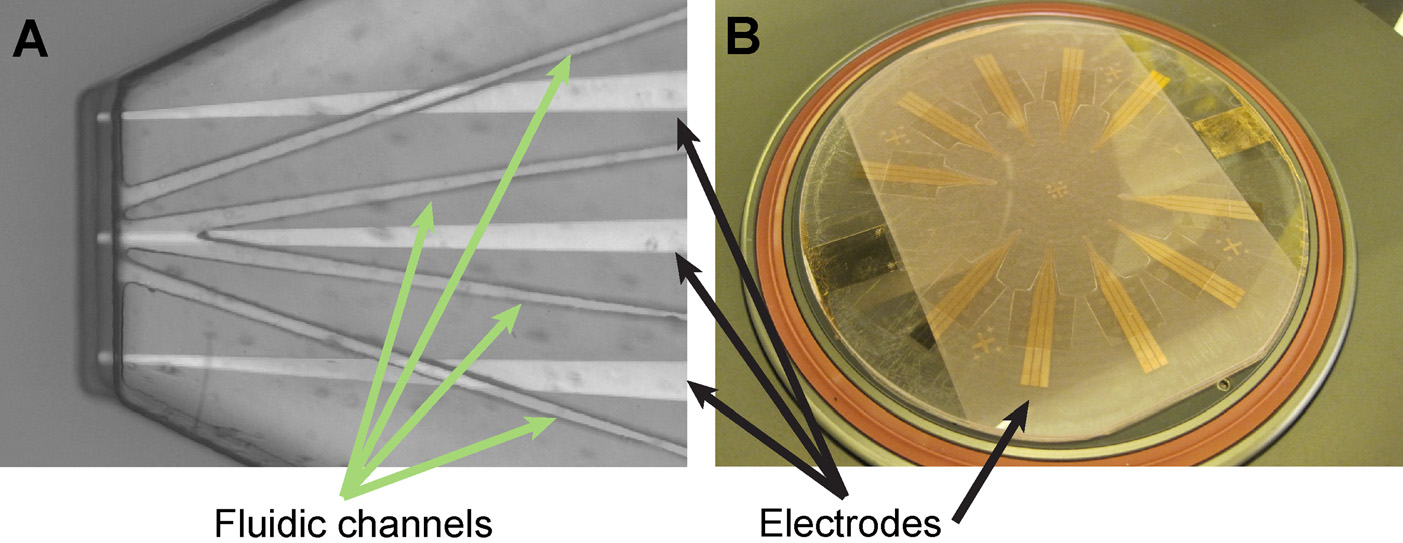
Functionalized SU-8 probe for impedance sensing. (a) Microscope image of a tip, the green and the black arrows indicate the fluidic channels and the electrodes, respectively. Scale bar 100 *μ*m. (b) Photograph of the 4″ wafer after processing.

A microphotograph of the integrated SU-8 micropipette is shown in Figure 9(a), with gold electrodes aligned to the microfluidic channels. Figure 9(b) shows a wafer scale tip batch. To ensure precise fabrication and arrangement of the electrode structures due to the level of sensitivity needed, they were patterned in a radial arrangement in the center of the wafer. A schematic illustration of a free-standing dielectric impedance probe measurement is shown in Figure 1(b), with the field lines of simulations performed in COMSOL Multiphysics software. For proof-of-concept, impedance spectroscopy electronics was developed and integrated with the holding interface, and measurements were performed on microbeads as primitive cell model. Note that the objects of interest need to be located on substrates featuring a conductive surface (in our experiments Au, with a thickness of 25 nm for transparency. The details of these experiments can be found in the supplementary material S5.

### D. SU-8 probes with perforated side walls for microdialysis

Continuous monitoring of biological events both *in vivo* and *in vitro* is of high interest in neurophysiology and pharmacology. One indispensable technique that offers high temporal resolution is microdialysis.^37^ Conventional microdialysis probes are simple tubular membranes that surround a channel guiding a perfusate, which are typically inserted into tissues. Compounds of interest of a particular size, which is defined by the molecular cut-off of the membrane, can diffuse through the membrane in both directions. Diffusion follows the concentration gradient established between the probe-internal fluid and the external environment, i.e., delivering or removing substances is equally possible without net fluid exchange.^38^

**FIG. 10.**
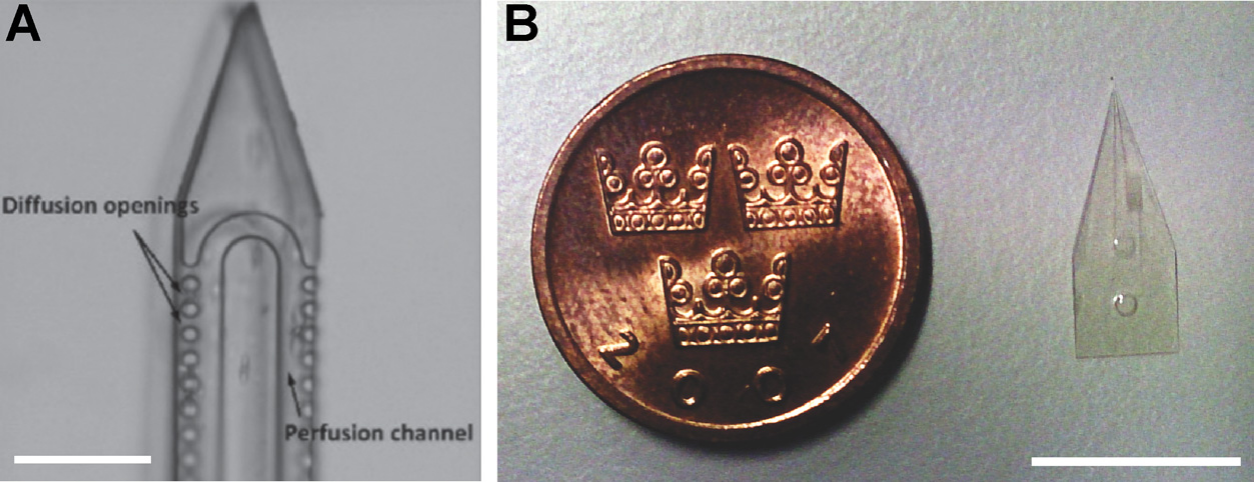
(a) Microphotograph of the probe tip, scale bar 100 *μ*m and (b) photograph of the SU-8 probe with perforated walls for microdialysis, in relation to the size of a common object. Scale bar 10 mm.

We used the SU-8 tip fabrication route to produce a microdialysis probe head, featuring perforated sidewalls, implemented as an array of pillars (Figure 10(a)). The inter-column spacing, or micropore size, can in principle be further reduced to the practically achievable resolution limit of SU-8, which would, however, even under best conditions still not be compared favorably with the conventional membrane material. The large pore size, compared to the conventional membranes, was chosen in order to eventually allow for embedding of hydrogel or other nanoporous material in the inter-column space, so that the probe essentially functions as a microfluidic scaffold for membrane material. Figure 10(b) shows an image of the entire probe with its fluidic ports.

Some reports on conventional microdialysis probes show recovery rates of around 100% with flow rates of about 100 nl/min.^39^ This is of course at the expense of the temporal resolution due to relatively large fluidic volumes being collected. The use of microfluidics and microfabricated probes allows for smaller flow rates to be achieved, while maintaining the temporal resolution due to smaller dead volumes, minimizing the Taylor dispersion. In our case, the finite element simulations described earlier were performed in order to determine the number of pores required to achieve a recovery rate of ∼50%. Analyte recovery in microdialysis is described by the total mass transfer of sampled substance across the probe, i.e., the ratio of the concentration of the analyte in the perfusate and the concentration of the analyte in the extracellular space. High recovery rates are obtained when the perfusate is pushed at low flow rates, allowing more time for the analyte to diffuse across the membrane at the expense of temporal resolution. The corresponding flow rate inside the channel was set to 0.18 nl/min. This represents the lower achievable limit, as the pump used to generate the pressure difference between the inlet and outlet of the device with its given channel length dimensions is practically limited to a minimum of 10 mbar. The expected recovery rate could be improved further by increasing the effective dialysis length by introducing more pores (openings in between the pillars). In our case, the number of openings intended to hold the membrane material (to provide the specificity or a certain cut-off) is 36 (18 on each side of a perfusion channel). The reason for not increasing this amount to further improve the recovery is due to the mechanical stability of a probe during the proposed dry release method, as further increasing the length of the needle-like tip may lead to its breakdown.

Critical fabrication challenges encountered during the integration of micropore structures into the side wall, such as planarization defects, collapse, and de-bonding, are discussed in the supplementary material S6.

### E. Notes

It is noteworthy that, even though SU-8 is generally considered a biocompatible material, the base of SU-8 is bisphenol A, which is a known endocrine disruptor.^40^ For this reason, the long-term usage of the material, for example, in implants or transdermal injection applications for human use, requires careful consideration, and further studies. In addition, although SU-8 does not pose any visualization issues within the conventional optical microscopy due to its relatively high optical transparency, it does, however, display autofluorescence within the 500–600 nm range. This could be a disadvantage in fluorescence microscopy studies.

## IV. CONCLUSIONS

A new wafer-scale microfabrication process for the generation of SU-8 microfluidic probes was designed, integrating a mechanical release step, to achieve free-standing final devices. We investigated the limits of the fabrication process, and adapted it to allow for incorporation of additional structural features and electric components.

We have constructed the fluidic prototype interfaces for supplying solutions to the fluid ports of the fabricated SU-8 devices, which also include implementations of all required external functional components, including electronic circuitry. The integrated wells in the PDMS-SU-8 composite device in the first interfacing strategy offer clear advantages over the external reservoir/supply line interface, such as very low dead volumes and confinement of all fluids within the device, which avoids contamination of tubes and manifolds. The tape-bonding technique we used for assembling the hybrid device is still rather cumbersome, as it requires a special tape, such as the double-sided silicon rubber bonding tape #5302A by Nitto Denko. Processes for irreversible bonding of SU-8 to PDMS have been reported,^41,42^ and they might be of use to further optimize and scale up the process.

We consider SU-8 a suitable material for fabricating a variety of functional microchannel devices with high mechanical rigidity, chemical stability, and exactly defined shape and size. The SU-8 processes described in this paper allow particularly for facile production of batches of microfluidic open-volume superfusion devices with well-defined tip-geometry, which in process effort and required time, and particularly in application features, compare favorably to PDMS processes and devices.

## V. SUPPLEMENTARY MATERIAL

See supplementary material for details of the microfabrication process, additional data, and probe designs.
